# Identifying potential patient-specific predictors for anterior cruciate ligament reconstruction outcome – a diagnostic in vitro tissue remodeling platform

**DOI:** 10.1186/s40634-020-00266-2

**Published:** 2020-07-04

**Authors:** Marc van Vijven, Bart van Groningen, Joyce N. Kimenai, Maria C. van der Steen, Marina van Doeselaar, Rob P. A. Janssen, Keita Ito, Jasper Foolen

**Affiliations:** 1 Orthopaedic Biomechanics, Department of Biomedical Engineering, Eindhoven University of Technology, Building 15, Groene Loper, Gemini-Zuid 4.12, PO Box 513, 5600MB Eindhoven, The Netherlands; 2 Institute for Complex Molecular Systems, Eindhoven University of Technology, Eindhoven, the Netherlands; 3 Department of Orthopaedic Surgery, Máxima MC, Eindhoven, the Netherlands; 4 Department of Orthopaedic Surgery, Catharina Hospital Eindhoven, Eindhoven, the Netherlands; 5 Fontys University of Applied Sciences, Eindhoven, the Netherlands

**Keywords:** Anterior cruciate ligament, Hamstring tendon autograft, In vitro micro-tissue platform, Patient-specific cell properties, Tissue remodeling

## Abstract

**Purpose:**

Upon anterior cruciate ligament (ACL) rupture, reconstruction is often required, with the hamstring tendon autograft as most widely used treatment. Post-operative autograft remodeling enhances graft rupture risk, which occurs in up to 10% of the patient population, increasing up to 30% of patients aged under 20 years. Therefore, this research aimed to identify potential biological predictors for graft rupture, derived from patient-specific tissue remodeling-related cell properties in an in vitro micro-tissue platform.

**Methods:**

Hamstring tendon-derived cells were obtained from remnant autograft tissue after ACL reconstructions (36 patients, aged 12–55 years), and seeded in collagen I gels on a micro-tissue platform. Micro-tissue compaction over time – induced by altering the boundary constraints – was monitored. Pro-collagen I expression was assessed using ELISA, and protein expression of tenomodulin and α-smooth muscle actin were measured using Western blot. Expression and activity of matrix metalloproteinase 2 were determined using gelatin zymography.

**Results:**

Only micro-tissues corresponding to younger patients occasionally released themselves from the constraining posts. Pro-collagen I expression was significantly higher in younger patients. Differences in α-smooth muscle actin and tenomodulin expression between patients were found, but these were age-independent. Active matrix metalloproteinase 2 expression was slightly more abundant in younger patients.

**Conclusions:**

The presented micro-tissue platform exposed patient-specific remodeling-related differences between tendon-derived cells, with the micro-tissues that released from constraining posts and pro-collagen I expression best reflecting the clinical age-dependency of graft rupture. These properties can be the starting point in the quest for potential predictors for identifying individual patients at risk for graft rupture.

## Background

The anterior cruciate ligament (ACL) connects the femur to the tibia and is one of the most important ligaments for knee joint stability. It consists of a predominately anisotropic collagen I extracellular matrix populated by fibroblast-like cells [24]. Upon acute overloading (trauma), the ACL can rupture, with a prevalence of 100,000–200,000 per year in the United States [9]. A ruptured ACL impairs knee stability, can impede the patient’s normal activity levels, and even lead to work-related disabilities. A completely torn ACL does not self-regenerate, and therefore, in 50% of the rupture cases an ACL reconstruction is required [18], often performed using a hamstring tendon (e.g., semitendinosus/gracilis tendon) autograft [28, 51].

After ACL reconstruction with an autograft, a remodeling process is initiated that transforms the tissue to match its new (mechanical) environment [16]. This remodeling can roughly be divided in an early graft healing phase, a proliferation phase and a ligamentization phase [18, 38, 41]. The early graft healing phase with central graft necrosis, hypocellularity and no detectable revascularization – resulting in nutrient deprivation – occurs in approximately the first 4 weeks after surgery. The necrotic fibroblasts secrete cytokines (e.g., tumor necrosis factor-α, interleukin 6, vascular endothelial growth factor (VEGF)) and matrix metalloproteases (MMPs) [55]. The latter mainly comprise collagenases (MMP1, MMP8 and MMP13) and gelatinases (MMP2 and MMP9), which break down the collagen extracellular matrix gradually [48]. The proliferation phase covers approximately the second and third month after surgery. VEGF-induced graft vascularization supplies cell nutrients, and combined with the cytokines secreted in the early graft healing phase, this stimulates myofibroblasts – characterized by α-smooth muscle actin (αSMA) expression – from the synovial fluid to invade the graft. The myofibroblasts produce more collagen III relative to collagen I, and deposit this more isotropically. In this phase, MMP production is high as well, which combined with the isotropic collagen III seriously impairs graft strength [18, 38, 41]. The ligamentization phase can last up to 2 years after surgery and is characterized by (partial) recovery of the fibroblastic cell phenotype. The cells align with the main loading direction, deposit anisotropic collagen I and therefore graft strength increases towards – but never fully attains – that of the native ACL [17, 18, 38, 41]. It can take up to 2 years after surgery for patients to return to their pre-injury activity level [33], which is ultimately achieved by approximately 65% of recreational athletes [3].

Early return to sports, traumatic re-injury, concomitant knee instabilities, surgical errors and/or impaired graft strength during remodeling – particularly the proliferation phase – can lead to ACL graft rupture, which occurs for up to 10% of the patients, and for patient younger than 20 years this increases even up to 30% [13, 14, 27, 33, 52]. This age-dependency of graft rupture risk could be ascribed to larger participation in (risk-taking) activities by younger patients, but biological differences in the tissue remodeling process may also contribute [27, 52]. Besides age, biological differences in tissue remodeling may also be related to gender [21], and which hamstring tendon is used for the reconstruction: an adequate autograft can usually be made of the semitendinosus tendon, but sometimes the gracilis tendon needs to be added [12, 20], and variations in regenerative capacity between both tendons have been observed [34]. Currently, it remains unknown whether graft rupture risk can be predicted from the biological properties of the resident fibroblasts-like cells in the graft. Therefore, the aims of this research were: 1) to assess various patient-specific tissue remodeling-related properties of human hamstring tendon-derived cells (TDCs; obtained from remnant autograft tissue after ACL reconstructions) in an in vitro micro-tissue remodeling platform; and 2) to relate the remodeling-related properties of patient-derived cells to patient age, gender and tendon type (semitendinosus or gracilis tendon), in order to identify cell properties that could potentially predict graft rupture.

## Methods

### Harvesting and expanding tendon-derived cells

Remnant tissue fragments from the most proximal and/or distal part of semitendinosus and/or gracilis tendons were collected after Institutional Review Board approval (METC N16.148) and processed immediately after ACL reconstructive surgery. The tendon fragments were disinfected with 70% ethanol and washed repeatedly with sterile phosphate buffered saline (PBS). The tissue was cut into small pieces and the extracellular matrix was dissolved in a sterile-filtered 2 mg/ml collagenase IV (Gibco, 17,104,019) solution in Dulbecco’s PBS (DPBS; Sigma-Aldrich, D4031) at 37 °C. After 6–22 h (depending on the tissue volume as well as time of day of surgery), the solution was centrifuged for 5 min at 1100 rpm, and the supernatant was discarded. The pellet containing cells and small tissue fragments was resuspended in medium consisting of high-glucose Dulbecco’s Modified Eagle Medium (HG-DMEM; Gibco, 42,430–025) and Nutrient Mixture F-12 Ham (F12; Gibco, 21,765–029) in a 1:1 (v/v) ratio, supplemented with 20% fetal bovine serum (FBS; Sigma-Aldrich, F7524) and 5% penicillin-streptomycin (pen-strep; Lonza, DE17-602E), and cultured for 7 days at 37 °C and 5% CO_2_ with 2–3 medium changes. Subsequently, the pen-strep concentration was lowered to 1%, and TDCs were cultured until cryopreservation after reaching sub-confluency in passage 1 or 2. In total, TDCs were successfully harvested from 41 tendons obtained from 36 patients (Table 1; Table S-1 for a complete overview).

**Table 1:** Demographics and origin of tissue fragments

		tendons	patients
**total**	total (#)	41	36
	semitendinosus	30	
	gracilis	11	
**male**	total (#)	23	20
	semitendinosus	18	
	gracilis	5	
**female**	total (#)	18	16
	semitendinosus	12	
	gracilis	6	
**age**	average (y)	27.7	27.9
	std. deviation (y)	12.9	12.9
	maximum (y)		55
	minimum (y)		12

### Micro-tissue culture, compaction and image processing

**Fig. 1:**
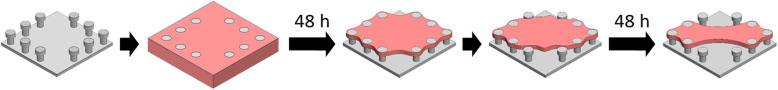
Schematic overview of the experimental procedure in the micro-tissue platform. To the array of micro posts (grey), a suspension of cells and collagen (pink) was added. Cells contracting and applying force to their collagenous environment results in formation of a predominantly isotropic micro-tissue around the constraining posts in the subsequent 48 h. By releasing the tissue constraints of the four outermost posts, a tissue remodeling process is initiated that results in further compaction into a more anisotropic tissue during the next 48 h

After cryopreservation, TDCs were grown to sub-confluency in growth medium consisting of HG-DMEM and F12 (1:1) with 10% FBS and 1% pen-strep and seeded in a micro-tissue platform as described before [7]. Briefly, systems of Dragon Skin™ (Smooth On, Dragon Skin™ 10 SLOW) posts were cast in 6-well plates (Fig. 1), disinfected with 70% ethanol, and treated with 0.2% Pluronic® F-127 (Sigma-Aldrich, P2443) in MilliQ for 1 h, to make surfaces non-adhesive. Afterwards, systems were washed with sterile MilliQ and UV-sterilized. TDCs were suspended at 1.5 million cells/ml in 71% (v/v) growth medium, 25% rat tail collagen I (Advanced Biomatrix, 5056) and 4% 1 M NaOH (Merck, 106,498) in PBS. 60 μl cell suspension was seeded in each system (Fig. 1), and after 45 min gelation at 37 °C, 4 ml growth medium, supplemented with 0.25 mg/ml L-ascorbic acid-2-phosphate sesquimagnesium (Sigma-Aldrich, A8960), was added to each well.

After 48 h, micro-tissues had formed around the posts due to collagen gel compaction by the TDCs. The constraints of the four outermost post were released, inducing further micro-tissue compaction (Fig. 1), which was monitored using brightfield imaging in a Leica DMi8 microscope with incubation box at 37 °C and 5% CO_2_. An image was made every hour, for 48 h. Micro-tissues in additional well plates that could not be monitored simultaneously were imaged immediately after releasing the constraints and 48 h later using an EVOS™ XL Core microscope (ThermoFischer Scientific). Micro-tissues that were still constrained by the remaining posts after 48 h were snap-frozen and stored at − 80 °C until further use in the follow-up analyses described below. Micro-tissues that had released from the remaining posts were not processed for further analyses. For a few patients, the number of micro-tissues was too low to perform all follow-up analyses or repeat an unsuccessful read-out. The number of patients included in each analysis is presented in Figs. 3, 4, 5 and 6.

**Fig. 2:**
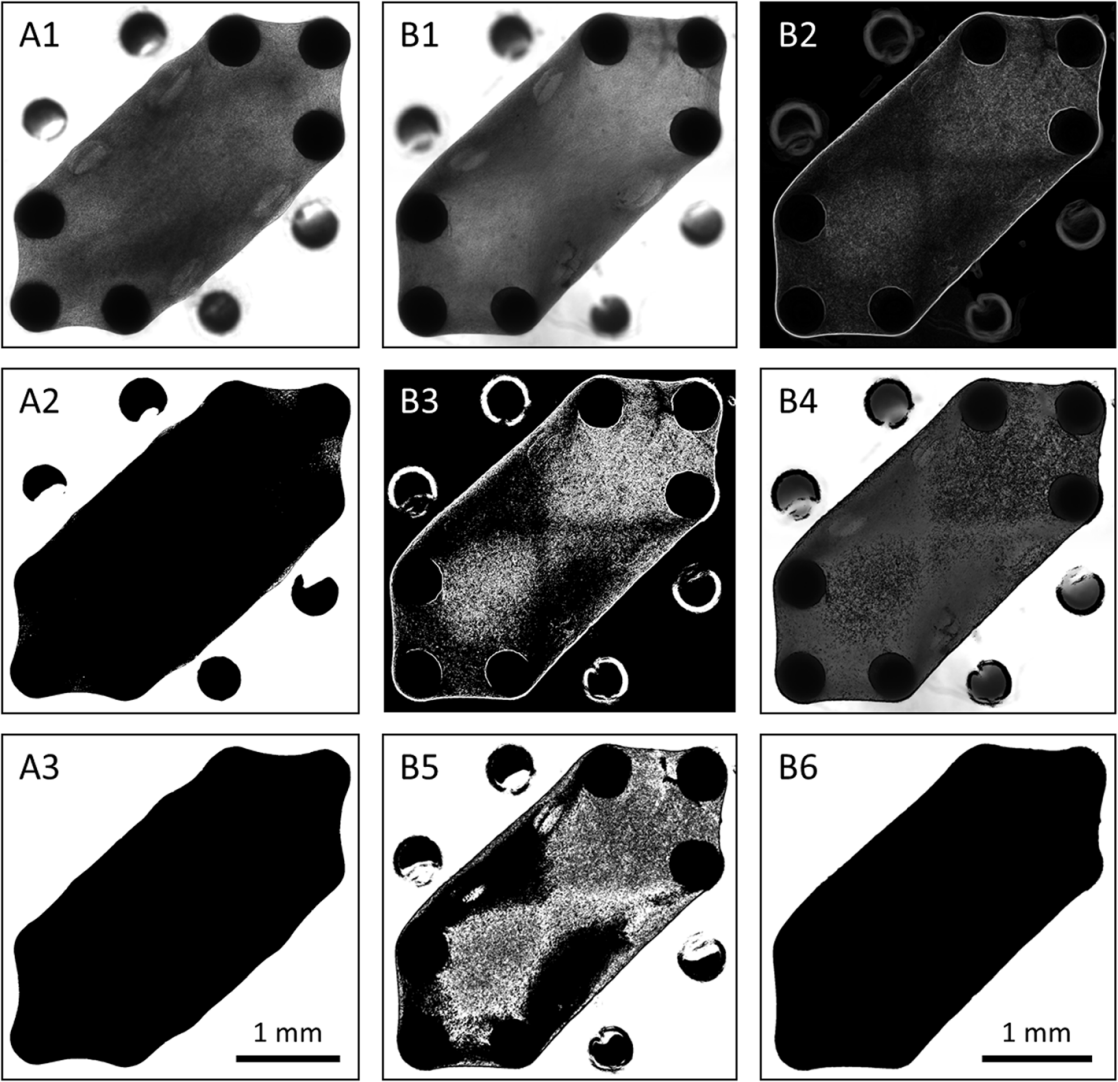
Steps to obtain top-view micro-tissue surface area. (A) Based on absolute intensity values: the original image (A1) was binarized (A2), the largest connected component was selected, and any remaining holes were filled (A3). The object size (in pixels) was counted and converted to the absolute surface area. In case strategy A failed, strategy B was applied. (B) Based on micro-tissue: on the original image (B1) edge detection was performed (B2). Image 2 was binarized (B3), and subtracted from the original image (B4). The resulting image was binarized (B5), the largest connected component was selected, and any remaining holes were filled (B6)

Microscopy images were analyzed using a custom-made Matlab (MathWorks) script calculating the top-view surface area of the micro-tissues over time, based on a grey value threshold, which is illustrated in Fig. 2 strategy A. For images where the absolute intensity values were insufficient to identify the micro-tissue silhouette, edge detection was performed on the original images in ImageJ [42] before applying the Matlab script (Fig. 2 strategy B).

### Pro-collagen I ELISA

Collagen I production was assessed with ELISA, by means of human pro-collagen I expression. One snap-frozen micro-tissue per remnant hamstring tendon tissue was lysed in 100 μl RIPA buffer (Sigma-Aldrich, R0278) on ice, and sonicated. Pro-collagen I expression was assessed using the Human Pro-Collagen I alpha 1 Matched Antibody Pair Kit (Abcam, ab216064), according to the manufacturer’s instructions, and quantified using the included pro-collagen I alpha 1 standard.

As a sample loading control, samples were semi-quantitatively tested for similarity of cell lysate contents using Western blot for housekeeping protein tubulin (following the method in the section ‘Western blot’), and outliers were defined as more than 1.5x the inter-quartile range below the first quantile or above the third quantile [19].

### Western blot

For protein expression analysis of tendon marker tenomodulin (37 kDa), myofibroblast marker αSMA (42 kDa) and housekeeping protein tubulin (50–55 kDa), one snap-frozen micro-tissue per hamstring tendon tissue was lysed as described above. Lysed samples were mixed 1:1 (v/v) with Laemmli buffer (composition in Supplementary material) and 20% (v/v) 100 mM 1,4-dithiothreitol (Sigma-Aldrich, D0632) in MilliQ, and denatured for 5 min at 98 °C. Samples were loaded in 10% polyacrylamide gels (Biorad, 1,610,158) (composition in Supplementary material) and separated by size using gel electrophoresis at 15 mA per gel for approximately 1.5 h.

Afterwards, proteins were transferred overnight to a blotting membrane, at 4 °C and 25 V. The membrane was blocked for 30 min with 50 mg/ml milk (Campina, Elk skimmed milk powder) in PBS/0.1% Tween (Sigma-Aldrich, 8.22184), followed by overnight incubation at 4 °C of the primary antibodies: rabbit anti-tenomodulin (Abcam, ab203676; 1:4000), rabbit anti-αSMA (Abcam, ab5694; 1:2000) and rat anti-tubulin (Novus Biologicals, NB600–506; 1:4000) in 50 mg/ml milk in PBS/0.1% Tween. The blot was incubated with the secondary antibodies: goat anti-rabbit HRP (Abcam, ab6721; 1:20,000) followed by rabbit anti-rat (Invitrogen, 61–9520; 1:20,000) in 5 mg/ml milk in PBS/Tween for 1 hour each. Finally, the blot was incubated for 2 min with Super SignalWest Dura Extended Duration Substrate (Thermofisher, 34,075), and visualized with the Isogen ProXima. Protein band intensities on the Western blots were determined using a custom-made script in Matlab.

### Gelatin zymography

For measuring MMP2 expression and activity using zymography, one snap-frozen micro-tissue per hamstring tendon tissue was lysed as for ELISA, and centrifuged for 10 min at 2000 rpm at 4 °C. The supernatant was mixed 2:1 (v/v) with sample buffer (composition in Supplementary material), loaded in 10% polyacrylamide/10% gelatin gels (composition in Supplementary material), and separated using gel electrophoresis at 120 V for approximately 1.5 h. The gel was permeabilized using 2.5% Triton X-100 (Millipore, 1.08603) in MilliQ for 2 × 30 min, and rinsed overnight in substrate buffer (composition in Supplementary material), both at 37 °C. Afterwards, the gel was stained for 2 h at room temperature while gently shaking, followed by 2 × 30 min de-staining (composition of both solutions in Supplementary material). Gels were visualized with the Isogen ProXima. Active MMP2 (62 kDa) and inactive MMP2 (pro-MMP2; 72 kDa) band intensities were determined as for Western blot and normalized to global (image) background and local (gel) background intensity. The active fraction was expressed as the active MMP2 protein band intensity divided by the total amount, e.g. active and inactive MMP2 band intensities combined. Gelatin zymography is also suitable to visualize MMP9 (92 kDa) protein bands, but these were not visible in our experiments.

### Statistical analysis

Statistical analyses were performed using R Commander [8]. For all read-outs normality was assessed with quantile-comparison plots. Multiple Regression Models were tested with age, gender and tendon type as independent variables and relative micro-tissue surface area after 48 h, pro-collagen I, tenomodulin, αSMA or active MMP2 protein expression or MMP2 active fraction as dependent variables. Similarly, for intact/released micro-tissues as dependent variable, a Logistic Generalized Linear Model was tested with age, gender and tendon type as independent variables. In all analyses, the significance level was set at α = 0.05.

## Results

### Micro-tissues corresponding to younger patients compact faster and release from posts more often compared to older patients

**Fig. 3:**
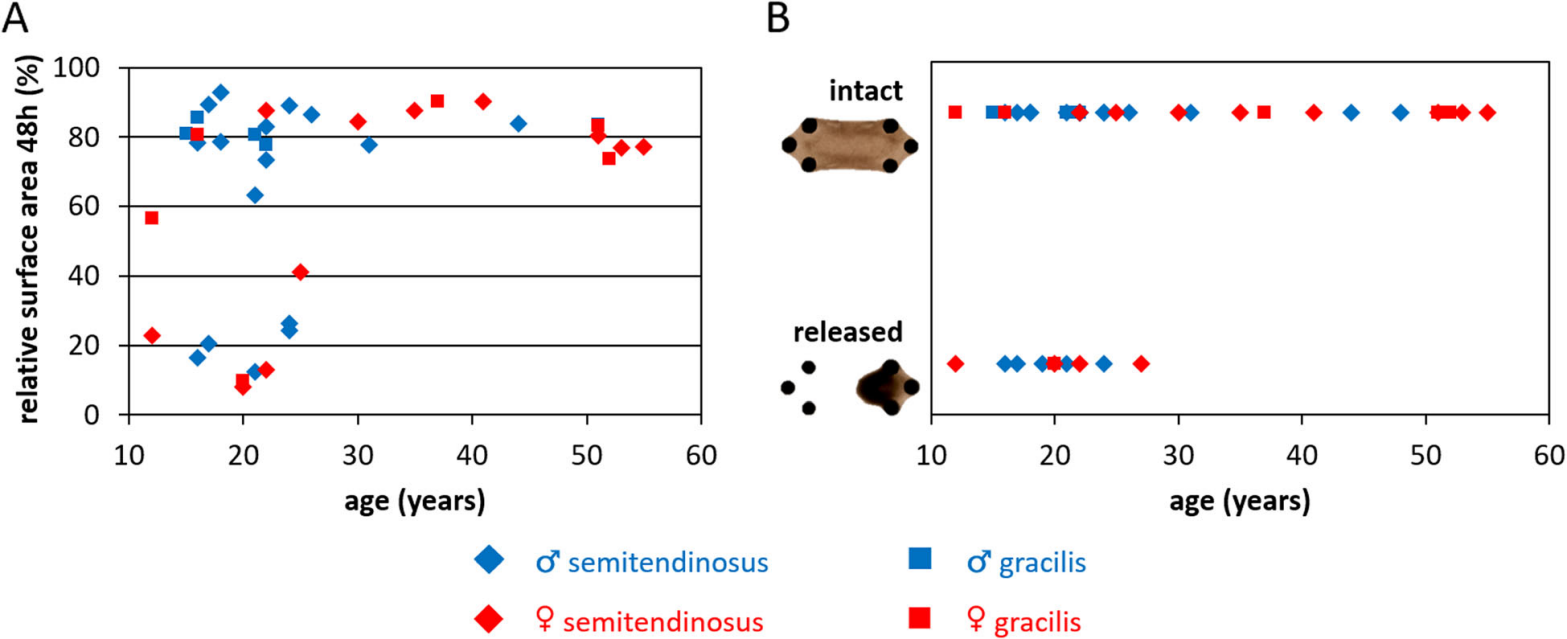
Micro-tissues representative for younger patients occasionally released from the posts during induced remodeling. **a** Top-view surface area after 48 h remodeling relative to 0 h, and **b** micro-tissues staying intact or releasing from remaining posts compared to patient age. Significant age-dependencies were detected for both the relative surface areas and micro-tissues staying intact versus those that released. *n* = 41

Micro-tissues were produced with cells from 41 tendons obtained from 36 patients. For 2 tendons (#7: ♀ 27y semitendinosus; #24: ♂ 19y semitendinosus) these micro-tissues released from the posts already before the constraints of the outermost posts were released. For both patients, cells from the gracilis were not available, and therefore these patients could not be included in further analysis. Top-view micro-tissue surface areas corresponding to the remaining 39 tendons are shown in Figure S-1, and the surface area after 48 h relative to 0 h is depicted in Fig. 3a. This relative surface area was significantly dependent on age (*p* = 0.012), but not on gender (*p* = 0.13) or tendon (*p* = 0.23) (Table 2).

**Table 2:** Results multiple regression models. ^a^ Pseudo-R^2^, since multiple R^2^ is not defined for logistic regression

read-out	multiple regression model coefficients									multiple R^**2**^
	age (years)			gender (♂ = 1)			tendon (semitend. = 1)			
	estimate	std. error	***p***-value	estimate	std. error	***p***-value	estimate	std. error	***p***-value	
**intact/released**	0.19	0.09	*0.042	1.6	1.0	0.10	−2.7	1.5	0.07	^a^ 0.30
**surface area 48 h (%)**	0.96	0.36	*0.012	15	9	0.13	−12	10	0.23	0.21
**pro-collagen I (ng/ml)**	−15	5	*0.01	145	144	0.32	−94	141	0.51	0.33
**tenomodulin / tubulin (−)**	0.0006	0.0012	0.64	−0.017	0.033	0.61	0.013	0.034	0.71	0.030
**αSMA / tubulin (−)**	0.023	0.034	0.50	1.1	0.9	0.24	−1.1	0.9	0.24	0.086
**active MMP2 (A.U.)**	−0.17	0.24	0.48	3.1	6.6	0.65	−0.4	6.7	0.95	0.039
**MMP2 active fraction (−)**	−0.0005	0.0011	0.64	−0.0009	0.031	0.98	−0.05	0.03	0.12	0.10

*p < 0.05

As can be observed in Fig. 3a and is indicated by the crosses (×) in Figure S-1, micro-tissues corresponding to 10 tendons from 9 patients released from the posts during the induced compaction, and the oldest patient this happened for was 27 years (Fig. 3b). Micro-tissues corresponding to the remaining 31 tendons from 27 patients remained intact over 48 h and were included in further analyses – as well as one single intact micro-tissue from patient #28. Whether micro-tissues were intact or released from the posts was significantly age-dependent (*p* = 0.042), but independent of gender (*p* = 0.10) or tendon type (*p* = 0.07) (Table 2).

### Pro-collagen I expression decreases with patient age

**Fig. 4:**
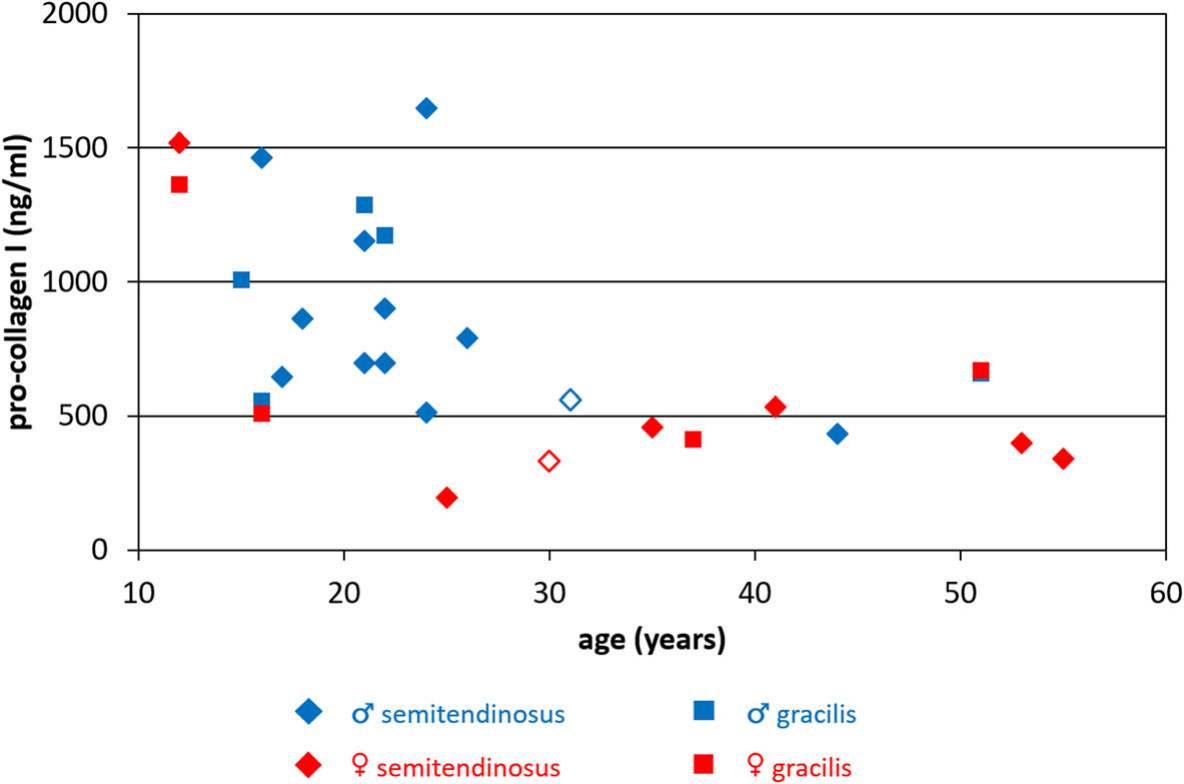
Pro-collagen I content decreased with increasing patient age, with an apparent patient cut-off age around 25 years. Patient-specific human pro-collagen I α1 content per micro-tissue was measured using ELISA and analyzed with respect to patient age. Outliers in cell lysate content are indicated by the open symbols . *n* = 28

To determine patient-specific production of collagen I by the TDCs, human pro-collagen I protein expression in the lysed micro-tissues was quantified using ELISA (Fig. 4). Semi-quantified cell lysate contents – by means of protein band intensities in Western blots for housekeeping protein tubulin – are shown in Figure S-2C. Identified outliers (Figure S-2D) are in Fig. 4 indicated by the open symbols. There were only outliers with a tubulin protein band intensity below average.

Pro-collagen I content was up to 5 times higher for younger patients and dropped for older patients with an apparent threshold around 25 years. The age-dependency of pro-collagen amount was statistically significant (*p* = 0.01), opposed to gender (*p* = 0.32) or tendon type (*p* = 0.51) (Table 2).

### Inter-patient variations in αSMA and tenomodulin protein expression are independent of age

**Fig. 5:**
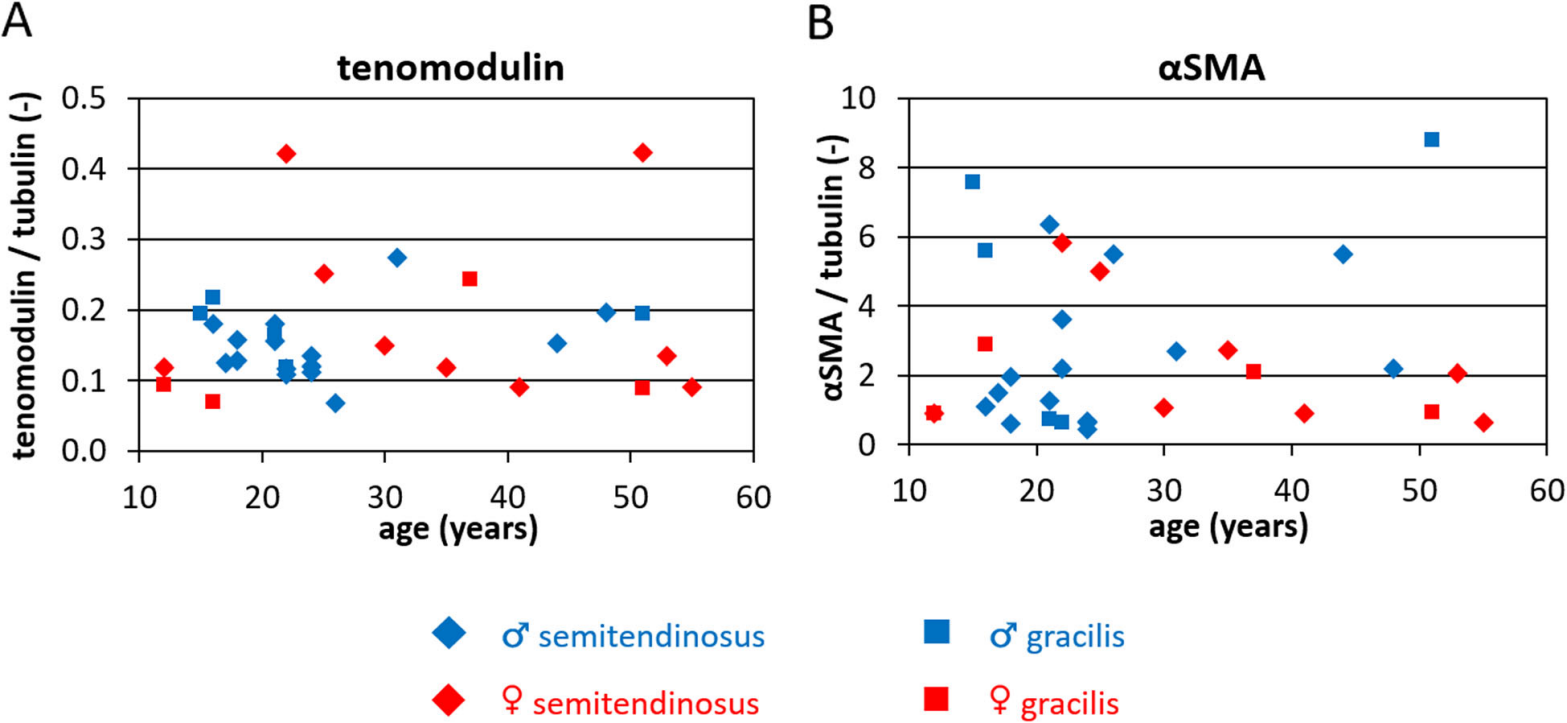
Large inter-patient differences in tenomodulin and αSMA expression were observed, although there was no significant age-dependency. Semi-quantitative protein expression of (**a**) tenomodulin and (**b**) myofibroblast marker α-smooth muscle actin (αSMA) were measured using Western Blot, and compared to patient age. *n* = 32

In order to investigate phenotypic properties of the TDCs in the micro-tissues, protein expression of tenogenic marker tenomodulin and myofibroblast marker αSMA were semi-quantitatively determined using Western blot. Western blot bands can be found in Figure S-2, and protein band intensities normalized to housekeeping protein tubulin are shown in Fig. 5.

Protein expression of tenomodulin (Fig. 5a) was remarkably low, with visible protein bands in only 2 samples, although an intensity signal was detected in the image analysis of all samples. Expression of αSMA (Fig. 5b) displayed up to 21-fold differences between patients. Interestingly, higher expression of stress fiber protein αSMA did not seem to be associated with faster or stronger compaction of the micro-tissues. No significant dependencies on age (tenomodulin: *p* = 0.64; αSMA: *p* = 0.50), gender (*p* = 0.61; *p* = 0.24) or tendon type (*p* = 0.71; *p* = 0.24) were observed for either of the proteins (Table 2).

### Active MMP2 is slightly more abundant in younger patients, whereas MMP2 active fraction shows little inter-patient variation

**Fig. 6:**
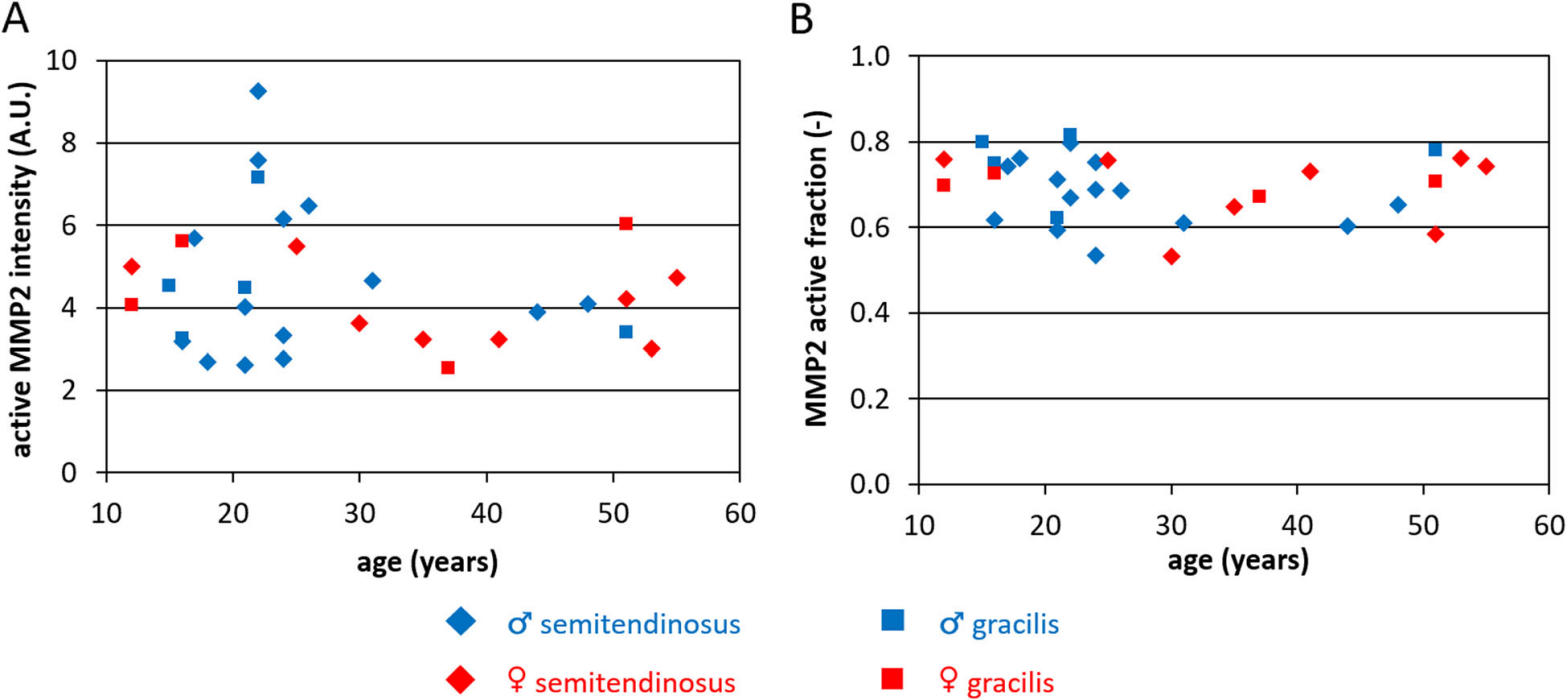
Active MMP2 was (non-significantly) more abundant in younger patients, whereas the active fraction MMP2 varied only little between patients. **a** Protein band intensities for active MMP2 on gelatinase zymography. **b** MMP2 active fraction, defined as the protein band intensity for active MMP2 divided by the total amount, e.g. active and inactive MMP2 protein band intensities combined. Both quantities were compared to patient age. *n* = 30

As a measure for matrix-degrading enzyme activity, MMP2 activity was determined using gelatinase zymography. MMP9 (92 kDa) protein bands were not visible. Zymograph bands of active MMP2 (62 kDa) and inactive MMP2 (pro-MMP2; 72 kDa) are depicted in Figure S-3, and resulting calculated active MMP2 intensities and active fractions are shown in Fig. 6a and b, respectively. Active MMP2 intensity was independent of gender (*p* = 0.65) or tendon type (*p* = 0.95), and appeared slightly higher in younger patients, but this trend was non-significant (*p* = 0.48). Only minor inter-patient differences in active fractions were observed, and significant dependencies on age (*p* = 0.64), gender (*p* = 0.98) or tendon type (*p* = 0.12) were absent (Table 2).

## Discussion

Reconstructive surgery upon ACL rupture – predominantly performed using the hamstring autograft – results in graft rupture in up to 10% of the patient population, increasing up to 30% of patients aged under 20 years. Inter-patient differences in tissue remodeling capacity, which are possibly age-related, can affect graft remodeling after surgery and therefore graft rupture risk. This creates opportunities for predicting patient-specific risk of graft rupture, but suitable predictors have not been identified yet. Therefore, the aims of this research were to assess patient-specific remodeling-related properties of patient TDCs from remnant tissue after ACL reconstructions with a hamstring tendon autograft, in an in vitro micro-tissue platform, and to identify potential patient-specific in vitro predictors for ACL graft rupture from these properties. For this purpose, tissue remodeling-related cellular properties of human hamstring TDCs were related to patient age, gender and type of hamstring tendon. TDCs were harvested from remnant tissue after ACL reconstructive surgeries and seeded in collagen I gels to form micro-tissues. After releasing micro-post constraints, the micro-tissues showed greater tissue compaction and even released from the remaining posts for some of the younger (≤27 years) patients. For the intact tissues, pro-collagen I, tenomodulin and αSMA expression, and MMP2 activity were determined. Pro-collagen I expression was significantly higher in cells from younger patients, with an apparent threshold around 25 years. Tenomodulin and αSMA expression varied between patients but were age-independent. Active MMP2 seemed slightly more abundant in younger patients, but MMP2 active fraction was almost equal for all patients. None of the read-outs was dependent on gender or tendon type.

Previous studies revealed intra-patient differences between the native ACL and hamstring tendon autograft before implantation, that converged after graft implantation [10, 23, 30, 46]. However, in order to identify potential predictors or risk factors for graft rupture, inter-patient variations of tissue properties need to be determined. To our knowledge, this is the first study comparing cellular properties of hamstring tendon autograft tissue from a large group of patients undergoing an ACL reconstruction, and relating this to patient age, which revealed potential predictors for graft rupture. A similar research was conducted for the outcome of rotator cuff repair [37], where cell growth and collagen production were age-dependent, whereas gender had no effect. However, no correlation between cell properties and clinical outcome of the rotator cuff repair was detected.

Releasing post constraints from the in vitro system induced micro-tissue remodeling and compaction via an altered equilibrium of cellular contraction and extracellular matrix stiffness [39]. The fact that some micro-tissues released from the remaining posts indicates that those cells were more contractile, and remarkably, this only occurred for cells from younger (≤27 years) patients. Reduced tendon (cell) contractility with age has been described before in rodents [25, 26], and was mostly ascribed to higher numbers of (αSMA-expressing) myofibroblasts in the tissue [53], but αSMA expression was not associated with faster or more compaction in the present study. Increased cell contractility in vivo would intuitively increase graft tension. Higher initial graft tension provides better patient outcomes – although too high tension can over-constrain the joint [6]. However, a link between tension and graft rupture risk has not been described. Still, cell contractility in vitro might have predictive value for in vivo outcome.

Pro-collagen I expression by the TDCs in the micro-tissues – as a measure for collagen production – was significantly age-dependent, decreasing up to 5-fold for patients older than 25 years. Literature on this subject is inconclusive, reporting both increasing and decreasing collagen content or production with age, as well as absence of age-dependency, in tendons and other tissues [22, 29, 36, 47, 50]. Nevertheless, the age-related pro-collagen I expression in this research did reflect the age-dependency of graft rupture rates as reported in literature [52], but it seems counterintuitive that graft rupture risk may increase with higher collagen production. We speculate though, that higher collagen production can induce fibrosis formation, when synthesis exceeds degradation [54]. This seems plausible to occur in certain younger patients, considering an expected average balance between collagen synthesis and degradation in healthy remodeling [1], as well as the reported non-significant age-dependency of active MMP2 expression and almost constant MMP2 active fraction. Besides that, faster collagen turn-over might be at the expense of collagen organization and therefore reduce mechanical strength [35].

The phenotypic properties of the TDCs in the micro-tissues were determined by means of tenomodulin and αSMA protein expression. However, neither of the protein expression levels was dependent on age, gender or tendon type. Particularly the independency of age, in contrast to the age-dependency of in vivo graft rupture described in literature, probably excludes these protein expression levels as in vitro predictor for in vivo graft rupture risk. It should be noted here that non-physiological in vitro culture conditions (i.e. high serum levels, hypercellularity and random cell morphology) can induce phenotypic drift of TDCs away from the tenogenic lineage [1, 49].

Statistically non-significant variations in active MMP2 protein band intensities following a decreasing trend with patient age were observed, implying inter-patient differences in protein expression. However, MMP2 active fraction was found almost equal in all patients. In previous research, increasing MMP2 expression [15, 56] and activity [31] with age were described in tendon and non-tendon tissues. Those samples were however directly obtained from human tissue (including inter-patient variation), whereas the homologous extracellular environment in our micro-tissues and presence of MMP2 in serum [4] may have evened out these parameters.

Whether the independent biological factors patient age, gender and tendon type (semitendinosus vs. gracilis) from this research are risk factors for in vivo graft rupture has been investigated before. Lower age is a well-known risk factor, and whereas the threshold is mostly set at 20 years [27, 52], intermediate re-rupture rates between 18 and 25 years have also been reported [43]. This matches the age-dependency we observed in pro-collagen I expression and micro-tissue release, with apparent thresholds around 25 and 27 years, respectively. Although female subjects are at higher risk of ACL rupture [45], this gender-dependency is not observed in graft rupture risk [40, 43], supporting our data where no gender-dependencies were detected in the biological properties of the TDCs from remnant autograft tissue. It has been reported before that use of a semitendinosus tendon alone or combined with a gracilis tendon as autograft does not affect in vivo graft rupture risk [40], in parallel to the absence of a significant difference between the tendon types in this study. The fact that remnant tissue from both the semitendinosus and the gracilis tendon was available for some patients, enabled intra-patient comparison of the cellular biological properties. Whether micro-tissues released from the posts or stayed intact was always equal for both tendons of the same patient. This underlined the patient-specificity of this read-out – independent of whether the semitendinosus tendon alone is used for the autograft or combined with the gracilis tendon – and consequently its potential as predictor for clinical outcome. For the other parameters, however, intra-patient variations were similar to inter-patient variations. This implies measurement variations originate from tissue-specific rather than patient-specific cell properties.

A few limitations and side notes of the current study need to be considered. Biological properties of the resident cells of ACL autografts were examined in this research, however, many of these cells are lost in the early graft healing phase of remodeling, and the graft properties are affected by the invading cells afterwards [32]. Therefore, it may be useful for future research to also investigate biological, remodeling-related properties of synovial fluid-derived cells and possibly identify predictors for graft rupture. Similarly, it should be noted that the high protein expression of αSMA in our system can be ascribed to trans-differentiation of the TDCs, whereas this in vivo mostly originates from invading myofibroblasts [32]. Before the cell isolation procedure, the risk for infection was reduced by briefly submerging the tissue in an ethanol solution. This treatment may have affected cell viability, but we believe this was limited to the tissue surface, since the time span for ethanol penetration into tendon tissue is in the range of hours [2]. The cell isolation procedure itself might have affected TDC properties too, but this approach was chosen since it enabled a wider screening of remodeling-related cell properties and fair comparison of the read-outs for all patients in a controlled manner. However, the micro-tissues that released from the posts were excluded from subsequent read-outs, because the time span without micro-post constraints – and therefore an altered mechanical environment for the cells – varied largely between patients, and ranged from 2 h to 37 h. This is in the same order of magnitude or exceeding the production rates of αSMA (approximately 2 h [44]), collagen I and MMP2 (both approximately 2–4 h [5, 11]). Therefore, the cells could have adjusted synthesis of these proteins to the new mechanical environment before the experiment was terminated, and the corresponding read-outs could be dominated by the unconstrained situation, potentially leading to an unfair comparison with the constrained micro-tissues. In follow-up research, the loss of micro-tissues for subsequent read-outs due to release from the constraining posts might be circumvented by including TDCs cultured only in a two-dimensional environment, but also techniques able to measure the identified potential predictors of re-rupture directly in the remnant patient tissue should be considered. That would eliminate successful TDC isolation from the patient inclusion criteria, prevent released micro-tissues to be excluded from subsequent read-outs, and therefore allow for a larger total number of patients included.

Altogether the complex process of in vivo graft remodeling cannot be completely mimicked in vitro in the proposed model system. However, the main criterium for a read-out with predictive value is a strong correlation of TDC properties with patient outcome – rather than mimicking the remodeling process. Despite the fact that more patients were included than in previous comparable studies [23, 37], re-rupture rates of up to 30% for younger patients and up to 10% for older patients which were reported in literature, translate into only a few possible clinical re-rupture cases that can be compared to our in vitro findings. Consequently, there might be insufficient statistical power to correlate in vitro read-outs to graft rupture. Therefore, correlations with other clinical outcomes such as knee stability examination by the orthopedic surgeon and patient reported outcome measures should be included.

## Conclusions

In conclusion, remodeling-related biological differences between patient TDCs could be assessed with the in vitro micro-tissue platform, and micro-tissues releasing from the posts and pro-collagen I expression reflected the age-dependency of re-rupture rates – as described in literature – best. Therefore, these read-outs can be the starting point in the quest for potential predictors for identifying individual patients at risk for graft rupture.

## Supplementary information

**Additional file 1: Figure S1.** Top-view surface areas over time. Top-view surface areas over time of all micro-tissues. Micro-tissues releasing from the posts are indicated with a cross (×).

**Additional file 2: Figure S2.** Western blot protein bands. (A) Protein bands in Western blot for αSMA and tubulin (housekeeping protein). (B) Protein bands in Western blot for tenomodulin and tubulin (housekeeping protein). (C) Protein bands in Western blot for tubulin to check cell lysate content of ELISA-samples. (D) Boxplot of semi-quantified tubulin protein band intensities, with identified outliers.

**Additional file 3: Figure S3.** Zymograph bands. Protein bands in gelatinase zymography for active MMP2 and inactive (pro-)MMP2.

**Additional file 4: Table S1.** Patient data.

**Additional file 5.** Compositions.

## Data Availability

The datasets used and/or analysed during the current study are available from the corresponding author on reasonable request.

## Abbreviations

ACL: Anterior cruciate ligament
αSMA: α-smooth muscle actin
DPBS: Dulbecco’s phosphate buffered saline
ELISA: Enzyme-linked immunosorbent assay
F12: Nutrient Mixture F-12 Ham
FBS: Fetal bovine serum
HG-DMEM: High-glucose Dulbecco’s Modified Eagle Medium
MMP: Matrix metalloproteinase
PBS: Phosphate buffered saline
pen-strep: Penicillin-streptomycin
TDC: Tendon-derived cell
VEGF: Vascular endothelial growth factor
