# Supplementary figures and images for: Identifying potential patient-specific predictors for anterior cruciate ligament reconstruction outcome – a diagnostic in vitro tissue remodeling platform
Source: J Exp Orthop. 2020 Jul 4;7:48. doi: 10.1186/s40634-020-00266-2 (PMC7335379; doi:10.1186/s40634-020-00266-2)

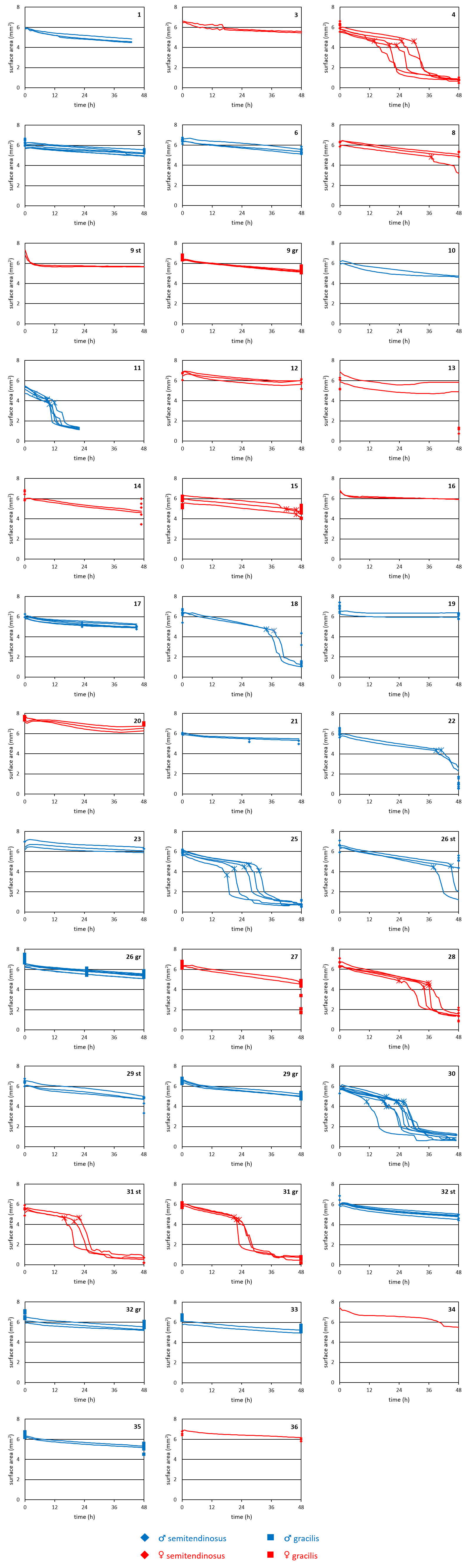

Supplement: Supplementary file 1 — Additional file 1: Figure S1. Top-view surface areas over time. Top-view surface areas over time of all micro-tissues. Micro-tissues releasing from the posts are indicated with a cross (×). [file 40634_2020_266_MOESM1_ESM.tif]

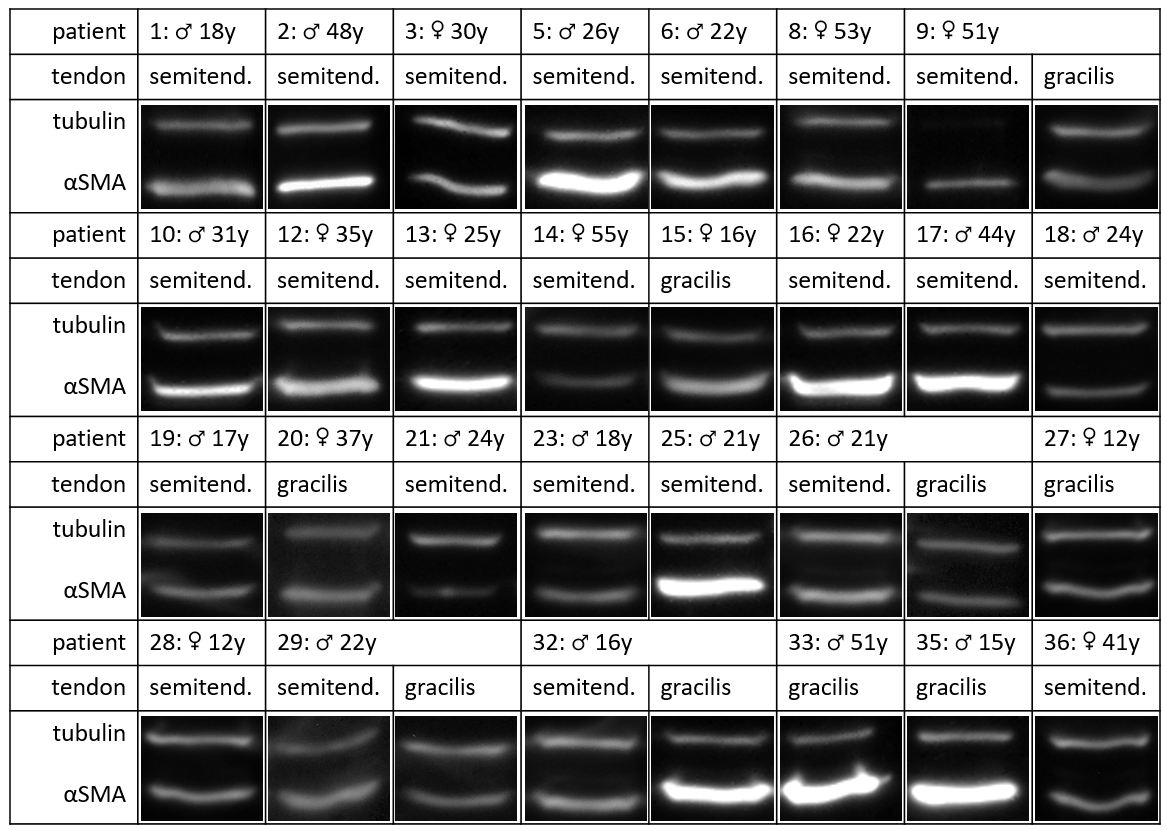

Supplement: Supplementary file 2 — Additional file 2: Figure S2. Western blot protein bands. (A) Protein bands in Western blot for αSMA and tubulin (housekeeping protein). (B) Protein bands in Western blot for tenomodulin and tubulin (housekeeping protein). (C) Protein bands in Western blot for tubulin to check cell lysate content of ELISA-samples. (D) Boxplot of semi-quantified tubulin protein band intensities, with identified outliers. [file 40634_2020_266_MOESM2_ESM.zip › Figure S-2a.tif]

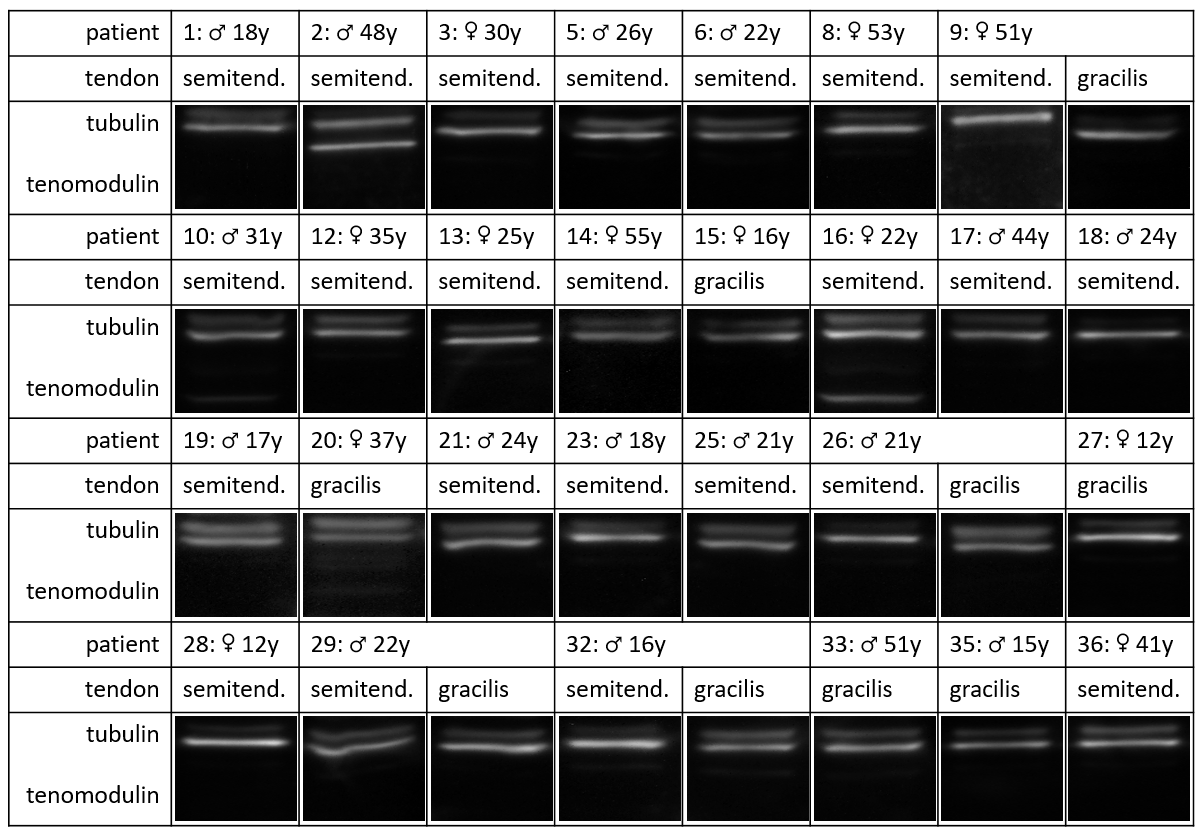

Supplement: Supplementary file 2 — Additional file 2: Figure S2. Western blot protein bands. (A) Protein bands in Western blot for αSMA and tubulin (housekeeping protein). (B) Protein bands in Western blot for tenomodulin and tubulin (housekeeping protein). (C) Protein bands in Western blot for tubulin to check cell lysate content of ELISA-samples. (D) Boxplot of semi-quantified tubulin protein band intensities, with identified outliers. [file 40634_2020_266_MOESM2_ESM.zip › Figure S-2b.tif]

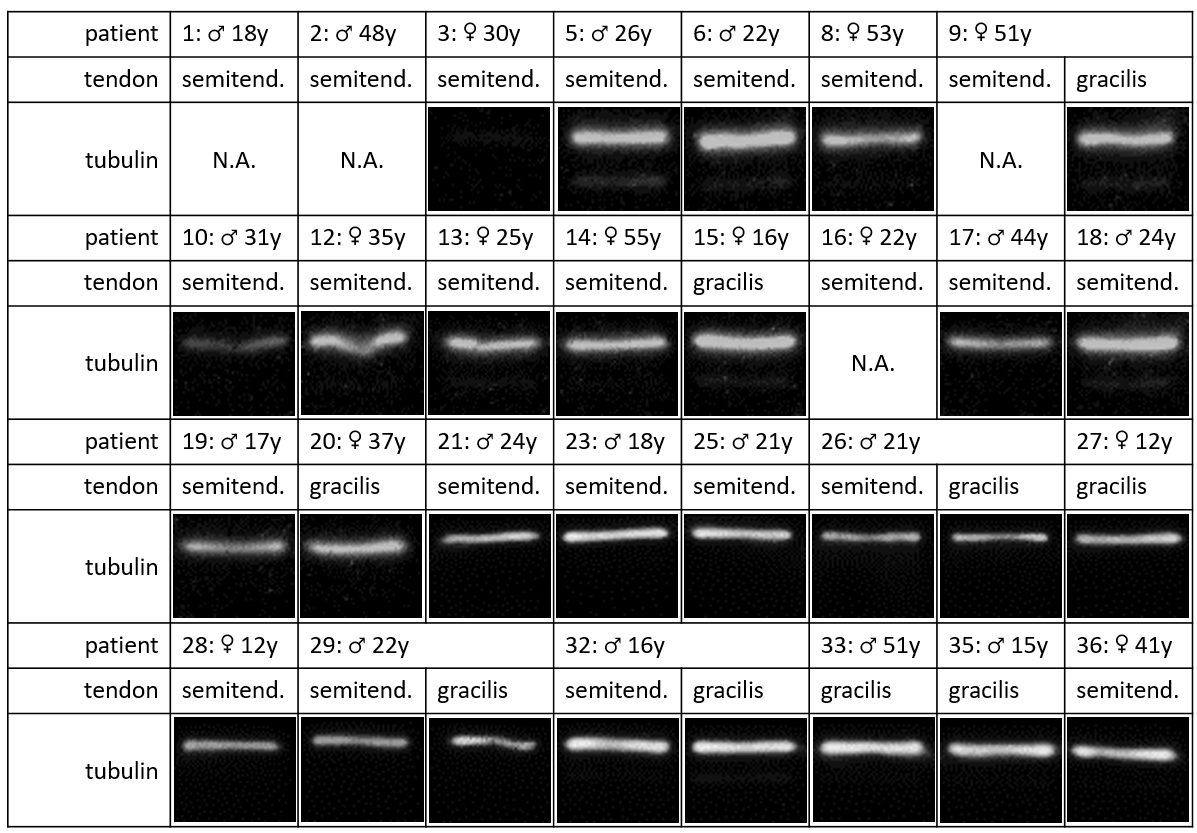

Supplement: Supplementary file 2 — Additional file 2: Figure S2. Western blot protein bands. (A) Protein bands in Western blot for αSMA and tubulin (housekeeping protein). (B) Protein bands in Western blot for tenomodulin and tubulin (housekeeping protein). (C) Protein bands in Western blot for tubulin to check cell lysate content of ELISA-samples. (D) Boxplot of semi-quantified tubulin protein band intensities, with identified outliers. [file 40634_2020_266_MOESM2_ESM.zip › Figure S-2c.tif]

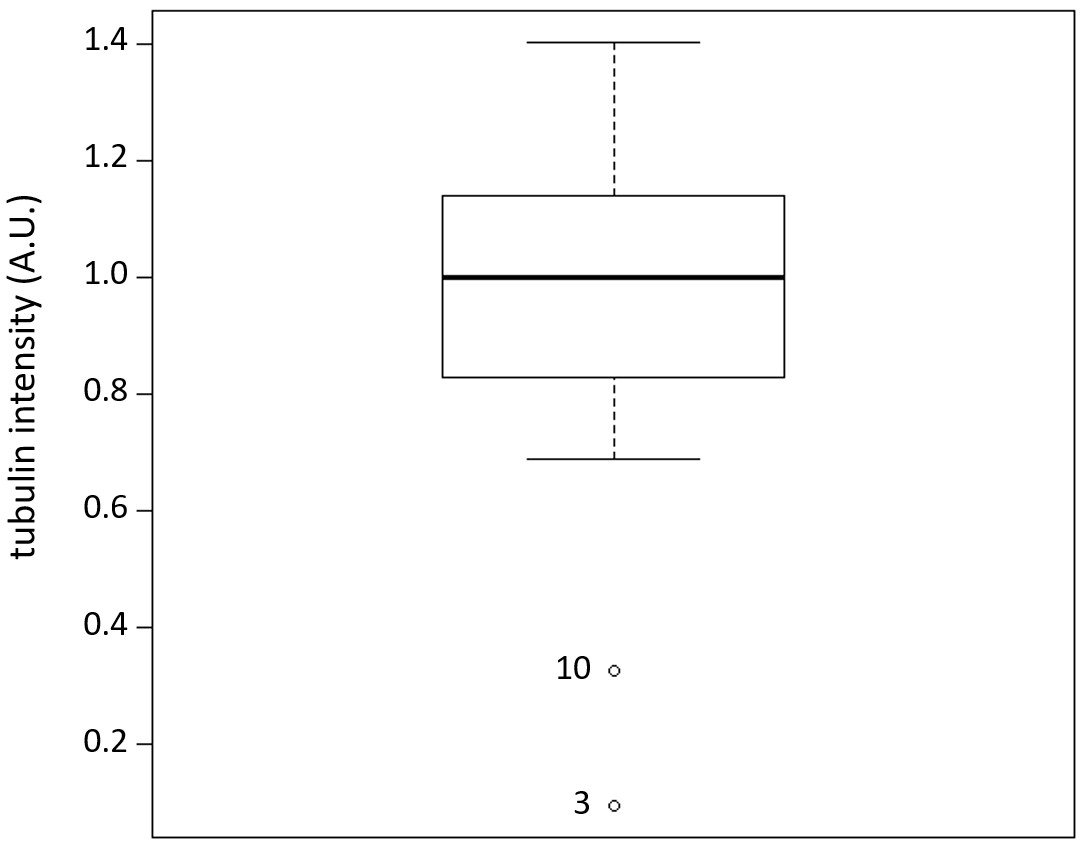

Supplement: Supplementary file 2 — Additional file 2: Figure S2. Western blot protein bands. (A) Protein bands in Western blot for αSMA and tubulin (housekeeping protein). (B) Protein bands in Western blot for tenomodulin and tubulin (housekeeping protein). (C) Protein bands in Western blot for tubulin to check cell lysate content of ELISA-samples. (D) Boxplot of semi-quantified tubulin protein band intensities, with identified outliers. [file 40634_2020_266_MOESM2_ESM.zip › Figure S-2d.tif]

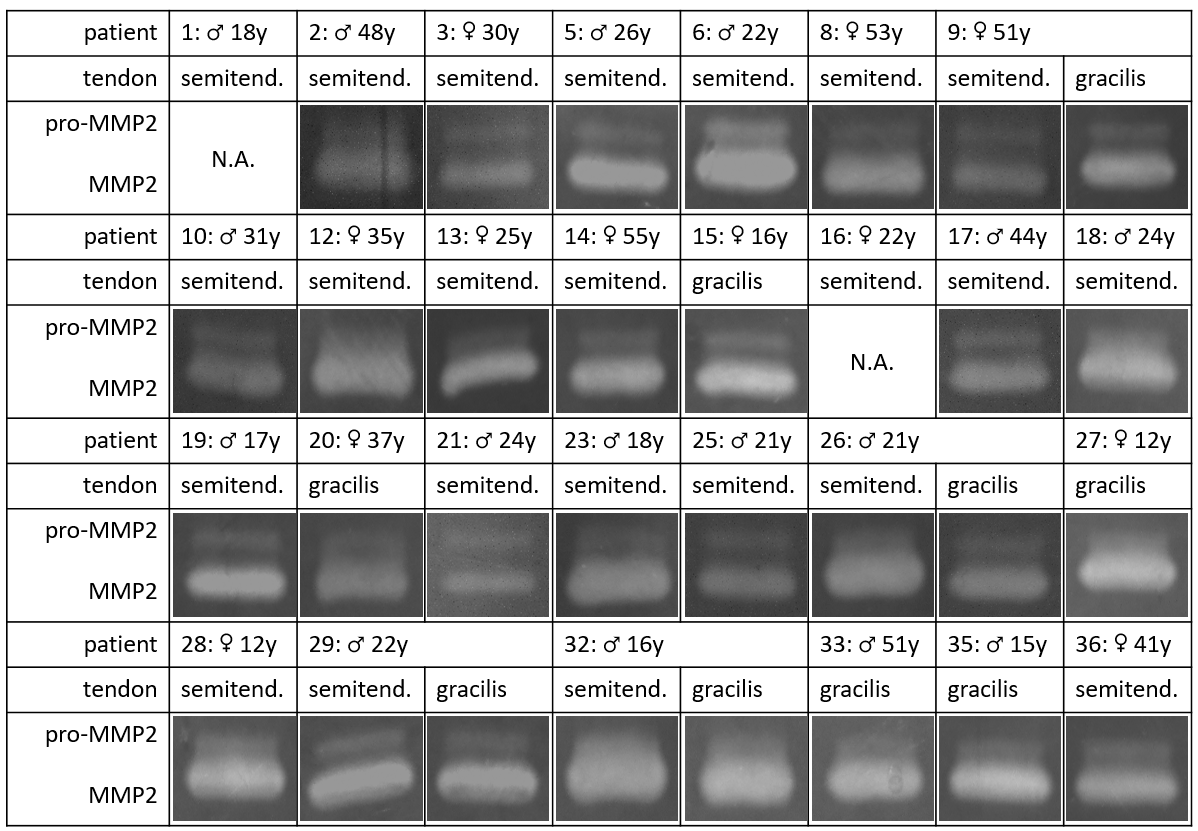

Supplement: Supplementary file 3 — Additional file 3: Figure S3. Zymograph bands. Protein bands in gelatinase zymography for active MMP2 and inactive (pro-)MMP2. [file 40634_2020_266_MOESM3_ESM.tif]
