# Supplementary material for: Identifying potential patient-specific predictors for anterior cruciate ligament reconstruction outcome – a diagnostic in vitro tissue remodeling platform
Source: J Exp Orthop. 2020 Jul 4;7:48. doi: 10.1186/s40634-020-00266-2 (PMC7335379; doi:10.1186/s40634-020-00266-2)
Supplement: Supplementary file 5 — Additional file 5. Compositions. [file 40634_2020_266_MOESM5_ESM.docx]

**Compositions**

***Gelatin zymography***

Gelatin/polyacrylamide running gel (per 100 ml)

25 ml 1.5 M Tris-HCl (Bio-Rad, 1610798)

33 ml 30% acrylamide/bisacrylamide solution, 37.5:1 (Bio-Rad, 1610158)

10 ml 6 mg/ml porcine gelatin (Sigma-Aldrich, G8150) in MilliQ

1 ml 20% sodium dodecyl sulphate solution (Sigma-Aldrich, 05030)

30 ml MilliQ

50 mg ammonium persulfate (Sigma-Aldrich, A9164) in 0.5 ml MilliQ

0.05 ml TEMED (Bio-Rad, 1610800)

Polyacrylamide stacking gel (per 100 ml)

25 ml 0.5 M Tris-HCl (Bio-Rad, 1610799)

16.7 ml 30% acrylamide/bisacrylamide solution, 37.5:1 (Bio-Rad, 1610158)

0.25 ml 20% sodium dodecyl sulphate solution (Sigma-Aldrich, 05030)

57.2 ml MilliQ

80 mg ammonium persulfate (Sigma-Aldrich, A9164) in 0.8 ml MilliQ

0.1 ml TEMED (Bio-Rad, 1610800)

Sample buffer (per 100 ml)

64 ml MilliQ

8.5 ml 20% sodium dodecyl sulphate solution (Sigma-Aldrich, 05030)

10.5 ml 1.5 M Tris-HCl (Bio-Rad, 1610798)

17 ml glycerol (Sigma-Aldrich, G7757)

3.4 g sucrose (Merck, 1.07687)

0.11 g bromophenol blue (Sigma-Aldrich, B5525)

Substrate buffer (per 100 ml)

0.61 g Tris (Merck, 1.08382)

0.054 g CaCl2 (Sigma-Aldrich, C2661)

100 ml MilliQ + hydrochloric acid (Merck, 1.00316)

adjusted to pH = 8.5

Staining solution (per 100 ml)

86 ml MilliQ

10 ml acetic acid (VWR, 2099.290)

4 ml methanol

0.1 g Brilliant Blue R 250 (Sigma-Aldrich, 27816)

De-staining solution (per 100 ml)

86 ml MilliQ

10 ml acetic acid (VWR, 2099.290)

4 ml methanol

***Western blot***

Polyacrylamide running gel (per 100 ml)

40.5 ml MilliQ

33 ml 30% acrylamide/bisacrylamide solution, 37.5:1 (Bio-Rad, 1610158)

25 ml 1.5 M Tris-HCl (Bio-Rad, 1610798)

0.5 ml 20% sodium dodecyl sulphate solution (Sigma-Aldrich, 05030)

100 mg ammonium persulfate (Sigma-Aldrich, A9164) in 1 ml MilliQ

0.04 ml TEMED (Bio-Rad, 1610800)

Polyacrylamide stacking gel (per 100 ml)

69.5 ml MilliQ

17 ml 30% acrylamide/bisacrylamide solution, 37.5:1 (Bio-Rad, 1610158)

12.5 ml 0.5 M Tris-HCl (Bio-Rad, 1610799)

1 ml 20% sodium dodecyl sulphate solution (Sigma-Aldrich, 05030)

100 mg ammonium persulfate (Sigma-Aldrich, A9164) in 1 ml MilliQ

0.1 ml TEMED (Bio-Rad, 1610800)

Laemmli buffer (per 100 ml)

1.21 g Tris (EMD Millipore, 1.08382) in 80 ml MilliQ + hydrochloric acid (Merck, 1.00316)

adjusted to pH = 6.8

20 ml glycerol (Sigma-Aldrich, G7757)

5 g sodium dodecyl sulphate (Sigma-Aldrich, L3771)

0.02 g bromophenol blue (Sigma-Aldrich, B5525)
